# Perspectives on Human Genetic Variation from the HapMap Project

**DOI:** 10.1371/journal.pgen.0010054

**Published:** 2005-10-28

**Authors:** Gil McVean, Chris C. A Spencer, Raphaelle Chaix

## Abstract

The completion of the International HapMap Project marks the start of a new phase in human genetics. The aim of the project was to provide a resource that facilitates the design of efficient genome-wide association studies, through characterising patterns of genetic variation and linkage disequilibrium in a sample of 270 individuals across four geographical populations. In total, over one million SNPs have been typed across these genomes, providing an unprecedented view of human genetic diversity. In this review we focus on what the HapMap project has taught us about the structure of human genetic variation and the fundamental molecular and evolutionary processes that shape it.

## Introduction

In human genetics, association studies aim to identify loci that contribute to disease susceptibility by comparing patterns of genetic variation between people with a disease (cases) and those without (controls) [1]. Without any prior knowledge about which genes are likely to be important, the researcher faces the expensive possibility of trying to look at all the 10 million or so polymorphic sites in the genome where the less common allele has a frequency of at least 1%, not to mention polymorphic inversions, duplications, microsatellites, and other forms of heritable variation. However, in recent years a number of empirical studies have revealed a structure to human genetic variation that could dramatically reduce the cost of association studies [2–9]. In particular, alleles at nearby loci often show strong statistical association (known as linkage disequilibrium [LD]). Coupled with observations that human recombination is concentrated into short (1–2 kb) hotspots that occur every 100–200 kb [10–12], and that these recombination hotspots are often coincident with a breakdown of allelic association [10], efficient genome-wide association studies became a possibility [13] because a few markers within each domain of strong association can be used to tag nearby variation. Here we use the term “tag” to imply that statistical tests for association carried out by using selected marker loci are as powerful (or nearly so) as if all single nucleotide polymorphisms (SNPs) were included.

However, in order to define efficient markers for subsequent studies, local knowledge of the structure of genetic variation across the genome is required. Choosing SNPs at set intervals across the genome, as one might in linkage studies, will fail to capture local patterns of allelic association and will consequently fail to tag efficiently. For this reason, the International HapMap Project was founded in 2002, with the goal of mapping the structure of allelic association across the human genome [14]. With the participation of funding agencies, academic research centres, and industrial partners in many countries, the initial aim was to genotype one SNP every 5 kb in the human genome across 270 individuals from four geographical populations. These individuals are 30 mother–father–offspring trios from the Yoruba people of Ibidan Peninsula in Nigeria (referred to as YRI), 30 such trios from the CEPH project in Utah (CEU), 45 unrelated individuals from the Han Chinese population of Beijing (CHB), and 45 unrelated individuals of Japanese ancestry from the Tokyo area (JPT) (for many analyses the CHB and JPT samples are combined within a single “analysis panel”). This project, referred to as the Phase 1 HapMap, is now complete, and the data, with associated summaries and query-based tools, are available online at http://www.hapmap.org with an accompanying manuscript published in Nature [15]. Further phases of the project, involving the typing of nearly 4 million SNPs across the same samples, and SNPs in a limited set of regions across multiple other population samples, are also under way.

What have we learnt from the project? For the medical geneticist the good news is that whole-genome association studies are still looking feasible. Technologies that provide high-throughput whole-genome genotyping of a few hundred thousand well-chosen SNPs should provide adequate power in most populations to detect single-locus associations for SNPs of moderate frequency and relative risk (we are being deliberately vague because the exact details depend on sample size and disease parameters [13]). Of course, not all complex diseases will have such an obvious genetic aetiology, and efforts to look for rare SNP effects [16], genetic interactions [17], or genotype-by-environment interactions [18] in candidate regions will no doubt also be fruitful. Furthermore, the design and analysis of association studies is still very much an area of active research that will only really be understood when large-scale association studies start becoming a reality.

However, while the use of the HapMap data for future association studies is the primary goal of the project, it also provides an unprecedented view of human genetic diversity that has provided novel insight into many other areas of biological interest. These include the distribution of recombination hotspots and coldspots, the effects of natural selection, and how these forces and others interact to shape human genetic variation. Our personal understanding of LD and how it relates to the underlying evolutionary and molecular forces has changed enormously through staring hard at more than a quarter of a billion genotypes. Therefore, what we are setting out to present in this review is a highly subjective set of observations made from the HapMap data that reflect what we have learned about the structure of human genetic variation.

## Understanding the Structure of Human Genetic Variation

Every chromosome carries a unique combination of alleles that is known as a haplotype. However, within regions of about 500 kb and less it is possible to find combinations of SNPs that are found in multiple unrelated individuals. Such “blocks” point to regions that have not been broken up by recombination and are often separated from each other by short regions where there is evidence for considerable recombination (recombination hotspots). These observations led to the idea of the human genome as a colourful mosaic of haplotype blocks delimited by recombination hotspots [19]. While this model is helpful in conveying the broad nature of human genetic variation, it fails to capture the true complexity. In this section we discuss four observations arising from analysis of the HapMap data that help to provide a more complete picture of the nature and causes of LD and genetic variation.

### 

#### In non-recombining regions, the genealogical tree determines the strength of LD.

Recombination acts to break down associations between alleles that arise because new mutations appear on a single genetic background. As we might expect, associations between alleles at loci separated by considerable genetic distances show consistently low levels of LD as measured by any statistic. However, and perhaps surprisingly, the converse is not true. Certain statistics of LD, and in particular the degree of statistical association between alleles as measured by the square of the correlation coefficient, *r* ^2^ [20], can take low values even in regions of low or no recombination (*r* ^2^ is the most relevant measure of LD for association studies because of the one-to-one relationship between *r* ^2^ and the relative power of statistical tests at a marker locus compared to the causative locus [21]).

**Figure 1:**
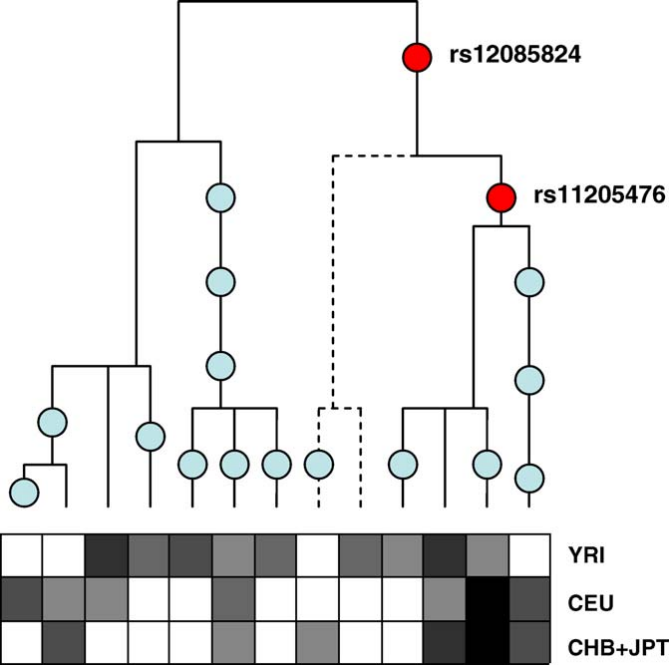
The Relationship between Genealogical History and Allelic Association The upper part of the figure represents the genealogy for the 13 haplotypes observed in a 40-kb region of Chromosome 1 (between SNPs rs12085605 and rs932087) where there is no evidence for recombination (for no pair of SNPs are all four possible combinations of alleles observed), with the location of polymorphic mutations indicated by circles. The lower part of the figure indicates the relative frequency of each haplotype in the sample from each of the three panels (in greyscale, with white indicating 0% and black indicating 100%). The dotted line in the genealogy indicates a branch of the tree that is not present in the CEU sample and whose removal results in perfect association between SNPs rs12085824 and rs11205476.

Why can LD be low even in non-recombining regions? When there is no recombination, all parts of the sequence share the same genealogical tree. So in terms of determining the strength of associations, what is important is where mutations appear in this tree (Figure 1). Two mutations that occur on the same branch of the genealogy will be present on the same chromosomes and, hence, will be in complete association. In contrast, two mutations that occur in completely different parts of the tree will occur in different chromosomes, and may only be weakly associated. This is really just another way of saying that the *r* ^2^ measure of LD is dependent on allele frequencies [22], but it has important consequences for association studies because the genealogical history of chromosomes taken from different parts of the world (or even repeat samples from the same places) are likely to be different.

We can see this effect in the example shown in Figure 1, a 40-kb region of chromosome 1. Here, we find 17 SNPs that show no evidence for recombination and result in 13 unique haplotypes that can be related to each other through a perfect phylogeny (i.e., there is no need to invoke repeat or back mutation). As one might expect, we observe differences in haplotype frequencies between panels, with the majority of haplotypes being found in only one panel (seven haplotypes are present in one panel only, compared to three being found in all). The difference in haplotype distribution leads to differences in allelic association; for example, SNPs rs12085824 and rs11205476 are in complete association in CEU (*r* ^2^ = 1), in strong association in CHB + JPT (*r* ^2^ = 0.88), and only moderately associated in YRI (*r* ^2^ = 0.58). More importantly, there is a clade of the genealogy (represented by the dotted line) that is not represented in the CEU sample (though it might be found with deeper sampling). Without this clade, the two SNPs effectively occur on the same branch and are therefore in complete association. The practical implication of this observation is that tagging choices may well be population specific, even in regions of low or no recombination. However, another more exciting possibility is that such differences between populations in genealogical trees constructed from non-recombining regions across the whole genome will provide novel insights into the demographic history of modern humans.

#### High-frequency haplotypes can cross recombination hotspots.

As stated above, within a population, associations between alleles separated by large genetic (recombination) distances are consistently low. But how large a distance is large? For example, is a single recombination hotspot sufficient to break down all associations? Put another way, if we are interested in tagging variation, should we break the genome into regions separated by recombination hotspots, or can tagging across hotspots ever be effective?

**Figure 2:**
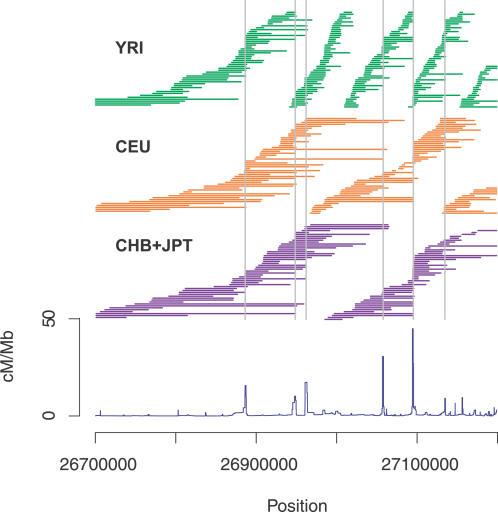
Patterns of Haplotype Structure and Recombination in the HapMap ENCODE Region on Chromosome 7q31.33 The estimated recombination rate (in centimorgans per megabase) is shown as a dark blue line, with statistically significant recombination hotspots (see [15] for details) as grey lines. For each analysis panel, each non-redundant haplotype with a frequency of at least 10% is represented by a horizontal line between the starting and ending SNPs (see [15] for details of methodology); the vertical height of these lines is arbitrary. Note that only one of the six hotspots is sufficiently strong to break all common haplotypes.

The answer is fairly straightforward. Recombination hotspots are rarely strong enough to remove all allelic association across them. Often, and particularly in the CEU, CHB, or JPT population samples, we find common haplotypes (at frequency of 10% and higher) that span recombination hotspots. Figure 2 demonstrates the relationship between common haplotypes and recombination rates in the ENCODE region on Chromosome 7q31.33 (data from [15]). As might be expected, haplotypes are considerably longer in CEU and CHB + JPT than in the YRI sample, reflecting the effect historical bottlenecks can have in reducing haplotype diversity and creating large haplotypes that take many hundreds of generations to be broken up by recombination. What is striking is that only one hotspot out of the six identified in the region is sufficiently hot to break all common haplotypes. Actually, we should not be particularly surprised by this result. At the hottest recombination hotspot identified across the autosomes, we would expect only one cross-over event in 114 meioses (a genetic distance of 0.9 cM), and at the “average” hotspot we would expect a recombination event every 1,300 meioses (0.075 cM).

#### Untaggable SNPs typically, but not always, occur in recombination hotspots.

No matter how hard you try, for certain SNPs there is just no other variant in the human genome that is in sufficient association to work effectively as its tag. Such “untaggable” SNPs are only problematic for association studies if you don't know where they are (otherwise they can just be included in genotyping studies). However, because even Phase II of the HapMap project will not type every SNP in the genome, it is important to learn about the distribution of such SNPs. In particular, can we predict where they might occur?

To answer this question we need to turn to the HapMap ENCODE project. This refers to a study within the project that resequenced 500 kb from each of ten ENCODE regions in 16 chromosomes from each analysis panel (i.e., a total of 48 chromosomes), followed by genotyping of all identified SNPs in the entire HapMap sample. While this does not provide complete ascertainment, it is expected to have identified almost all common (minor allele frequency > 5%) SNPs in each region (the average density of common SNPs is 1.5 per kilobase). A very high proportion of all common SNPs have at least one highly efficient potential tag (*r* ^2^ ≥ 0.8; 92% in CEU, 90% in CHB + JPT, and 80% in YRI), and the figures get better if you allow for less-efficient tagging and/or a higher threshold on minor allele frequency. However, across the ten ENCODE regions, a handful of really high frequency SNPs (minor allele frequency > 25%) have no tags at all (maximum *r* ^2^ < 0.2; 11/3,261 in CEU, 12/3,270 in CHB + JPT, and 20/2,961 in YRI).

**Figure 3:**
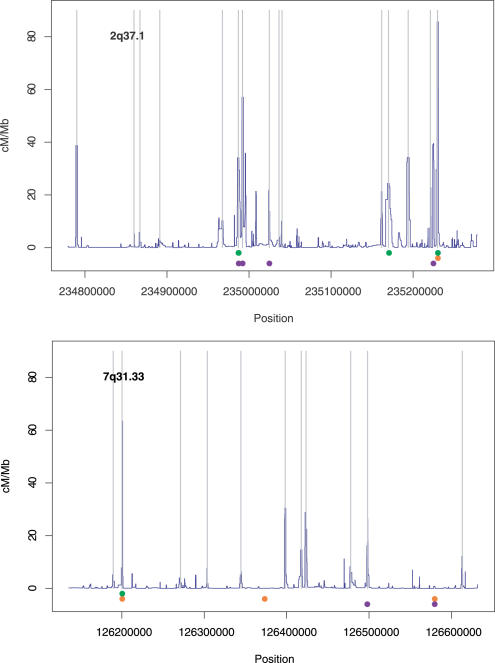
The Relationship between Recombination Rate, Recombination Hotspots, and the Location of Untaggable SNPs For two HapMap ENCODE regions the estimated recombination rate (dark blue line) and the location of statistically significant hotspots (grey lines) are shown along with the location of SNPs that are untaggable in the YRI (green) CEU (red), or CHB + JPT (purple) panels. Note that most, but not all, untaggable SNPs occur in recombination hotspots.

What might cause a really common SNP to be untaggable? One obvious possibility is that these SNPs lie in the middle of recombination hotspots. Figure 3 shows the location of the untaggable SNPs in two of the ENCODE regions, along with the estimated recombination rate profile. In the region on Chromosome 2q37.1, all untaggable SNPs fall in the middle of recombination hotspots. This is also true for two of the four untaggable SNPs in the region on Chromosome 7q31.33, but we need a different explanation for the other two in this region. One possibility is just chance. As seen above, even if there is no recombination, genealogical structure can lead to differences in allelic association between populations, and neither of these untaggable SNPs is completely untaggable in all populations. It is also possible that these SNPs might be hypermutable sites (such as methylated cytosine–guanine dinucleotides), or that they are hotspots of gene conversion, or that they have a high error rate (all of which would lead to low allelic association). Whatever the cause, the conclusion is that untaggable SNPs, while concentrated in recombination hotspots, are not restricted to them.

#### Regions of unusual genetic variation point to interesting biological features.

There is great heterogeneity across the genome in terms of patterns of genetic variation. Some of this heterogeneity is due to variation in factors such as mutation rate and recombination rate. Some of this heterogeneity arises because of the stochastic properties of mutation and genealogical history. But there are also other forces such as natural selection and genomic features such as inversions that may influence local patterns of variation. How can we look for the effects of such factors? There are two approaches. Either we can try to predict what we would expect to observe under models with and without such effects [23,24], or we can simply look at the empirical distribution of statistics of genetic variation and take as candidate regions those showing extreme or unusual patterns. The difficulty of the first approach is that accurately modelling human variation (and SNP ascertainment) is probably impossible. The difficulty of the latter approach is that there is no guarantee that empirically unusual patterns point to biologically interesting features.

However, it is possible to validate empirical approaches by asking whether regions where independent evidence points to biological interest are outliers in terms of genetic variation (or alternatively identify the statistics that identify such regions as unusual). The good news is that several genes or features for which biological interest is known do stand out as being unusual in the HapMap data in some sense. For example, the lactase gene (*LCT,* associated with lactose tolerance) has one of the highest relative extended haplotype homozygosity (rEHH [25]) scores in the CEU population, as does the beta-globin gene (*HBB,* associated with protection against *Plasmodium falciparum* malaria) in the YRI population. The HLA region (associated with resistance to multiple infectious diseases [26]) is one of a handful of gene clusters across the genome where there are haplotypes at frequencies of 1% across the combined population sample that span over 500 SNPs and more than 1cM. The known polymorphic inversion on Chromosome 17 [27] stands out as having the greatest number of SNPs in complete association (66 SNPs with *r* ^2^ = 1 in Phase I HapMap) in the entire genome, and there are only 33 nonsynonymous SNPs across the Phase I HapMap that show as much population differentiation as the SNP rs12075 typed in the Duffy gene (*FY,* associated with protection against *P. vivax* malaria). The implication of these findings is that other genomic regions with similarly unusual patterns of variation are candidates for biologically interesting loci. Of course, some may have such extreme statistics purely by chance, and genotyping projects are likely to miss certain features (such as high or low genetic diversity and rare mutations) that are informative about other biologically interesting loci.

Another question we can ask is whether genes previously reported as showing evidence for the action of historical selection (because they do not conform to the expectations of statistical models that assume neutrality) are also unusual within the empirical, genome-wide distribution. Table 1 shows the value of two selection statistics (Tajima's *D* [28] and Fay and Wu's *H* [29]) that are commonly used to infer the action of historical selection from genetic variation for 19 genes computed from the HapMap data (in 30-SNP windows around the midpoint of each gene). Because of the ascertainment bias in the frequencies of SNPs chosen for genotyping, we do not expect either statistic to follow the standard neutral distribution. However, we can ask whether these genes fall within the tails of the empirical distribution (computed from regions at least 30 kb from known genes) or within the tails of the empirical distribution of regions matched for local recombination rate (the variance of selection statistics is influenced by recombination rate such that more extreme values are expected in regions of low recombination [30]).

**Table 1:**
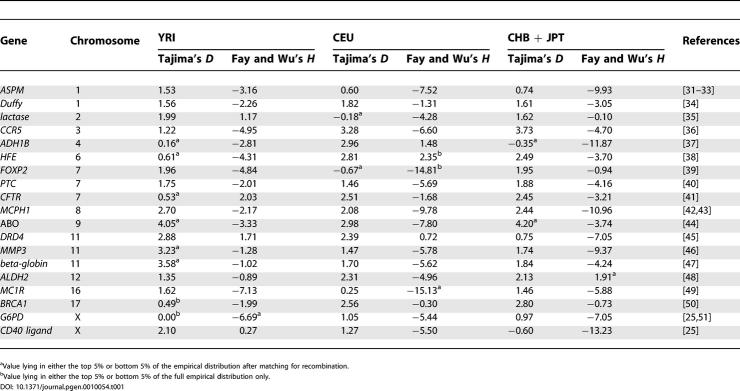
Selection Statistics in the HapMap Data for Genes Reported to Have Experienced Recent Adaptive Evolution

Of the 19 genes with previous evidence for historical selection, 12 show an unusual pattern of genetic variation in at least one population (defined as having a value lying in either the bottom 5% or top 5% of empirical values). Superficially, this result suggests that statistical tests based on rejecting a simple population genetics model are effective at detecting genes of interest. However, for 114 tests, we might expect 11 to lie in either the top or bottom 5% of observations, compared to the 17 observed. Another concern is that genes of known functional and selective importance, such as *Duffy* and *CD40 ligand,* do not fall in the tails of the empirical distribution of Tajima's *D* and Fay and Wu's *H* statistics and others, such as *MMP3, hemochromatosis (HFE),* and *aldehyde dehydrogenase 2 (ALDH2)* show patterns that are unusual, but not indicative of the action of recent selective sweeps.

There are two main conclusions from these analyses. First, that biologically interesting loci often do have unusual patterns of genetic variation, but that there is no single way of measuring “unusual” that is uniformly powerful for detecting the action of natural selection. Second, that rejection of neutral evolutionary models is no guarantee that the locus is unusual when compared to the rest of the genome. One of the great strengths of the HapMap data is that they will provide an alternative, empirical basis on which to assess how unusual the pattern of variation is at a given locus. However, it will still be many years before we know how reliable “looking in the tails” is as an approach to identifying genes of selective and functional importance.

## Conclusions

Integrating our knowledge about gene function, genome structure, chromatin organisation, recombination rate, mutation processes, and evolutionary history to provide a coherent understanding of the structure of the human genome and human genetic variation is a task that is just starting. It is also a task that has been greatly aided by the HapMap project with its unprecedented view of SNP variation, and there is no doubt that researchers will be uncovering fascinating patterns in the data for years to come. As the subsequent phases of the project progress, we can also expect to gain an even more detailed view of the differences between our genomes and the evolutionary and biological forces that have made us.

## Abbreviations

LD: linkage disequilibrium
SNP: single nucleotide polymorphism
